# CD36 mediates palmitate acid-induced metastasis of gastric cancer via AKT/GSK-3β/β-catenin pathway

**DOI:** 10.1186/s13046-019-1049-7

**Published:** 2019-02-04

**Authors:** Jiaomeng Pan, Zhiyuan Fan, Zhenqiang Wang, Qingqiang Dai, Zhen Xiang, Fei Yuan, Min Yan, Zhenggang Zhu, Bingya Liu, Chen Li

**Affiliations:** 1 Department of Surgery, Shanghai Key Laboratory of Gastric Neoplasms, Shanghai Institute of Digestive Surgery, Ruijin Hospital, Shanghai Jiao Tong University School of Medicine, Shanghai, 200025 People’s Republic of China; 2 Department of Pathology, Ruijin Hospital, Shanghai Jiao Tong University School of Medicine, Shanghai, 200025 People’s Republic of China

**Keywords:** CD36, Palmitate acid, Gastric cancer, Metastasis, AKT, GSK-3β, β-catenin

## Abstract

**Background:**

Gastric cancer (GC) has a clear predilection for metastasis toward the omentum which is primarily composed of adipose tissue, indicating that fatty acids may contribute to this phenomenon. However their function remains poorly understood in GC. In this study, we investigated the role of palmitate acid (PA) and its cellular receptor CD36 in the progression of GC.

**Methods:**

Immunohistochemical (IHC) staining was performed to detect CD36 expression in GC tissues and its clinical significance was determined statistically. CD36 over-expression and knock-down expression cell models were developed and tested in vitro. Wound-healing assays, migration assays, and invasion assays were performed and peritoneal implants into nude mice were done to assess the biological effects of PA and CD36. The underlying mechanisms were investigated using western blot, immunofluorescence (IF), quantitative real-time PCR (qRT-PCR) and antibody blocking assays.

**Results:**

PA promoted the metastasis of GC by phosphorylation of AKT, which facilitated the nuclear localization of β-catenin through inactivation of GSK-3β via phosphorylation. This tumor-promoting effect of PA was mediated by CD36, a cell surface receptor of fatty acids (FAs). The higher the CD36 expression levels in GC tissues correlated with the poorer the prognosis of patients according to the TCGA database, the GEO database and our own clinical data.

**Conclusions:**

Our experiments established CD36 as a key mediator of FA-induced metastasis of GC via the AKT/GSK-3β/β-catenin signaling pathway. CD36 might, therefore, constitute a potential therapeutic target for clinical intervention in GC.

**Electronic supplementary material:**

The online version of this article (10.1186/s13046-019-1049-7) contains supplementary material, which is available to authorized users.

## Background

Gastric cancer (GC) is the third leading cause of tumor-related death worldwide and is associated with great expense and suffering especially in eastern Asia mainly due to its high incidence of metastasis and recurrence [1–3]. Metastasis is one of the main factors influencing the prognosis of GC patients, and therefore, it is of great importance to investigate the underlying mechanism to improve treatments.

Fatty acid (FA) metabolism has recently been reported to play a vital role in the initiation and progression of multiple types of cancer both in vitro and in vivo [4–6]. FA metabolism involves uptake of exogenous FAs, lipid transport and storage and de novo synthesis of FAs. FAs include saturated FAs, monounsaturated FAs and polyunsaturated FAs. Studies of the effects of FAs on GC have shown that monounsaturated FAs, like oleic acid (OA), may promote GC migration and invasion [7], while polyunsaturated FAs, like docosahexaenoic acid (DHA), have been demonstrated to suppress GC’s metastatic properties [8]. However, the mechanism of the biological effects of saturated fatty acids on GC has not been illuminated and needs further investigation. Accumulating evidence has confirmed that CD36, a cell surface receptor of FAs, allows cells to take up lipids from the extracellular microenvironment and promotes FA oxidation (FAO) to produce ATP [9–12] potentially energizing tumor progression and metastasis [13–16]. Chen et al. [17] also reported that elevated expression of CD36 in GC tissues correlated with poor prognosis while the underlying mechanism remained elusive. Considering GC’s clear predilection for metastasis to the omentum, which is primarily composed of adipocytes [18] and the vital role of microenvironment in tumor progression, we put forward the hypothesis that FAs contribute to the metastatic cascade.

Here, we demonstrate that palmitate acid (PA), a long-chain saturated FA, promotes both the migratory and invasive abilities of GC cells by activating the AKT/GSK-3β/β-catenin signaling mediated by CD36 via increased uptake of exogenous PA. Furthermore, we found that increased expression of CD36 in tumor tissues was positively associated with depth of tumor local invasion, lymph node metastasis, higher TNM stage and poor prognosis of GC patients.

## Materials & methods

### Cell lines and reagents

GC cell lines, NCI-N87 and KATO-III, were purchased from American Type Culture Collection (ATCC). The GC cell lines HGC27, MKN45, MGC803, MKN28 and the immortalized normal gastric epithelial cell line, GES-1, were purchased from Shanghai Institute for Biological Sciences, Chinese Academy of Sciences. All cell lines were grown in RPMI-1640 medium (Gibco, BRL, San Francisco, CA, USA) supplemented with 5 μg/ml penicillin/streptomycin and 10% fetal bovine serum (Invitrogen, Carlsbad, CA, USA) in a humidified incubator at 37 °C with 5% CO_2_.

Docosahexaenoic acid (DHA) (Cat. # D2534, Sigma) was dissolved in ethanol according to Brown, M [19]. Palmitate acid (PA) (Cat. # P9767, Sigma), stearic acid (SA) (Cat. # S4751, Sigma), and oleic acid (OA) (Cat. # O1008, Sigma) were prepared as 2 mM stock solutions by dissolving in ddH_2_O at 70 °C, filtering through 0.4 μm and storing at 4 °C. The stock solutions were then added to 2% FA-free bovine serum albumin (BSA) medium made by dissolving FA-free BSA (Cat. # A7030, Sigma) in serum-free RPMI-1640 medium to a final concentration of 2% (*w*/*v*) and supplementing with 5 μg/ml penicillin/streptomycin to achieve the desired final FA concentration. The working concentration of the PI3K inhibitor, LY294002 (Cat. # S1105, Selleck) was 10 μM. The major antibodies used are listed in Additional file 1: Table S1.

### Clinical data and the cancer genome atlas (TCGA) and gene expression omnibus (GEO) database analysis

GC tissues and normal gastric tissues were collected from 250 patients who underwent gastrectomy between 1 September 2008 and 30 December 2013 in the Ruijin Hospital, Shanghai Jiao Tong University School of Medicine, Shanghai, China. None of these patients had received chemotherapy or radiation therapy before surgery. All samples from GC patients were confirmed by two experienced pathologists according to the seventh edition of the American Joint Committee on Cancer (AJCC) TNM staging system. After being fixed with formalin and embedded in paraffin, all samples were made into tissue microarrays (Outdo Biotech Co. Ltd., Shanghai, China). Among these 250 patients, we successfully followed up 84 patients for further prognosis.

GC gene expression data (mRNA, normalized RNAseqV2 RSEM) was retrieved from the TCGA database using the cBioPortal for cancer genomics for further analysis of the correlation between the expression level of CD36 and the AJCC TNM stage of patients with GC [20, 21]. The association between the expression level of CD36 and the prognosis of GC patients was analyzed online (http://www.oncolnc.org/ for the TCGA cohort and http://kmplot.com/analysis/index.php?p=background/ [Including GSE14210, GSE15459, GSE22377, GSE29272, GSE51105, GSE62254] for the GEO datasets, respectively) or in-house using our own cohort. To compare the mRNA level of CD36 between GC and normal tissues, we downloaded four microarrays containing data of cellular expression status of paired GC tissues and normal tissues from GEO datasets for further analysis (GSE63089, GSE38940, GSE84787 and GSE56807).

### Immunohistochemical staining

Immunohistochemical (IHC) staining was performed on tissue microarrays and peritoneal tumor modules according to a previously reported standard protocol used in the Shanghai Institute of Digestive Surgery [22]. The staining intensity was graded into four ranges (intensity score): no staining (0), light brown staining (1), brown staining (2) and dark brown staining (3). The number of positively staining GC cells was divided into four ranges (percentage score): < 5% (0), 5–30% (1), 31–70% (2), > 70% (3). The final staining score was calculated using the formula: overall score = intensity score × percentage score. A final score ≤ 3 was defined as negative staining, and > 3 as positive staining. The scores were evaluated by two independent, board-certified pathologists in an unbiased fashion.

### Western blot analysis

Cell samples were prepared in RIPA cell lysis buffer (Solarbio, Beijing, China) containing the protease inhibitor, phenylmethanesulfonyl fluoride. The concentration of protein was quantified using a bicinchoninic acid protein assay kit (Pierce, Rockford, IL, USA) based on a bovine serum albumin standard curve. A total of 20 μg protein was loaded onto a 10% sodium dodecyl sulfate polyacrylamide gel, the proteins were separated by electrophoresis and then transferred onto 0.22 μm PVDF membranes (Millipore, MA, USA). The membranes were blocked with 1 × TBST buffer containing 5% bovine serum albumin and incubated with the appropriate primary antibodies at 4 °C overnight. The antibodies involved are listed in Additional file 1: Table S1. HRP-conjugated secondary antibodies were purchased from Cell Signaling Technology and used at 1:5000 dilution. After exposure to secondary antibodies, membranes were washed and protein bands were visualized with Thermo Pierce enhanced chemiluminescent (ECL) western blotting substrate (Thermo, Waltham, MA, USA) and densitometry measurements were made using a Tanon 5200 gel imaging system (Tanon, Shanghai, China).

### Cell migration, invasion and wound-healing assays

Cell migration and invasion assays were performed using 24-well plates and 8 μm transwell inserts (Corning Life Science, Acton, MA, USA). For migration assays, 4 × 10^4^ tumor cells were suspended in 200 μl of serum-free RPMI-1640 medium containing either 2% BSA, 0.1 mM PA, 100 uM OA, 20 uM DHA or its control and cultured in the upper chamber. Fetal bovine serum (10%)-conditioned medium (700 μl) was added to the lower chamber of the 24-well plates. For invasion assays, the inserts were coated with Matrigel (50 μl/well) (BD Biosciences) and kept in a humidified incubator at 37 °C with 5% CO_2_ for at least 2 h before adding the cells. After a period of culture, the tumor cells remaining on the upper side of the inserts were removed with cotton swabs. The tumor cells that migrated through to the lower side of the inserts were fixed in methanol and stained with 0.5% crystal violet for 30 min at room temperature. Migrated cells were photographed with a Nikon Digital Sight DS-U2 (Nikon, Tokyo, Japan) camera attached to an Olympus BX50 microscope (Olympus Optical Co. Ltd., Tokyo, Japan). Five visual fields were randomly chosen to calculate the number of migrated cells. For the wound-healing assay, tumor cells were cultured in six-well plates until confluent, then scratched with a 20 μl pipette tip. The previous medium was replaced with fresh serum-free medium containing either 2% BSA, PA or SA every 12 h. Cells were photographed at 0 h, 18 h and 36 h after the scratches were made.

### Blocking assays

To specifically block the uptake of fatty acids by CD36, anti-CD36 (JC63.1) (Cat. # ab23680, Abcam, 1:500) was added with the IgA isotype (Cat. # ab37322, Abcam, 1:500) as control.

### Immunofluorescence (IF)

Cell samples were fixed in 4% paraformaldehyde at room temperature for 60 min. Sections were gently washed three times in 1× phosphate buffered saline (PBS) and then permeabilized with 0.25% Triton (Cat. # T9284, Sigma) in 1× PBS for 10 min. After being blocked with 3% bovine serum albumin for 1 h at room temperature, the sections were incubated with anti-β-catenin (Cat. # 8480, CST) primary antibodies for 1 h at room temperature and gently washed three times in 1× PBS. Sample sections were then incubated with Alexa Fluor 594-conjugated secondary antibody and DAPI (Cat. # 4083S, CST). Photos were taken using an Olympus BX50 microscope (Olympus, Tokyo, Japan).

### Plasmid construction and transfection

CD36 shRNA plasmids and negative control plasmids were constructed by Obio Technology (Obio Technology Co. Ltd., Shanghai, China) using the following target sequences: #1 (5′CCGACGTTAATCTGAAAGGA3′) and #2 (5′GAAGTTACATATTAGGCCAT3′) from Pascual, G [23]. The corresponding vector was pLKD-CMV-GPR-U6-Puromycin. MGC803 and HGC27 cells were cultured in six-well plates and transfected with 4 μg shRNA plasmids using Lipofectamine 2000 reagent (Cat. # 11668027, Invitrogen) following the manufacturer’s protocol. Cells were treated with puromycin (5 μg/ml) (Cat.# ANT-PR-5B, Invivogen) to produce stably transfected cells (MGC803/nc-shRNA, MGC803/CD36-shRNA, HGC27/nc-shRNA and HGC27/CD36-shRNA) for further study. The CD36 plasmid and its empty vector were kindly provided by Pascual, G [23]. The CD36 plasmid and corresponding empty vector were transfected into GC cells (MGC803, HGC27 and MKN28) using Lipofectamine 2000 following the manufacturer’s protocol. Stably transfected cells (MGC803/vector, MGC803/CD36, HGC27/vector, HGC27/CD36, MKN28/vector and MKN28/CD36) were selected by incubating them with puromycin (5 μg/ml). Knockdown and overexpression of CD36 were confirmed by western blot analysis.

### In vivo peritoneal tumor dissemination

Four week-old male BALB/c nude mice (Institute of Zoology, Chinese Academy of Sciences, Beijing, China) were housed in a pathogen-free room in the Animal Experimental Center, Ruijin Hospital, Shanghai Jiao Tong University School of Medicine, China. All animal experiments were performed in accordance with the official recommendations of the Chinese animal community. We randomly divided ten mice into two groups to investigate whether FAs could promote the growth of intraperitoneally implanted GC cells in vivo. One group of mice was fed with a 60 Kcal% high fat diet (Cat. # D12492i, Research Diets) and the other group of mice received normal diet for seven days before and after being intraperitoneally transplanted with 5 million MGC803 cells per mouse suspended in 150 μl PBS. Meanwhile, another twenty mice were randomly divided into four groups, all of which were fed with a 60 Kcal% high fat diet for seven days before and after being intraperitoneally injected with 5 million treated MGC803 cells (MGC803/nc-shRNA, MGC803/CD36-shRNA MGC803/Vector, MGC803/CD36, respectively) to identify whether the tumor-promoting effect induced by FAs depended on CD36 in vivo. All mice were euthanized under general anesthesia four weeks after injection and the peritoneal nodules were systematically observed.

### Fatty acids detection

Blood was drawn from mice, sera collected and a kit was used to assay for the concentration of long-chain FAs (Cat. # ab65341, Abcam) according to the manufacturer’s directions. Briefly, 2-50 μL of serum (volume adjusted to 50 μl/well with assay buffer) were added to 96-well plates in triplicate followed by 2 μl of ACS reagent per well. The plates were incubated for 30 min at 37 °C. Next, 50 μl of reaction mix was added to each well and the plates were incubated at 37 °C for 30 min protected from light. The absorbance at 570 nm was measured immediately afterwards on a microplate reader.

### Quantitative real-time PCR (qRT-PCR)

Total RNA was extracted with TRIzol reagent (Invitrogen) and cDNA synthesis was performed using a reverse transcription kit (Promega, Madison, WI, USA) according to the manufacturer’s instructions. The mRNA level of β-catenin was measured using the SYBR Green PCR Master Mix (Applied Biosystems, Waltham, MA, USA) and the Applied Biosystems 7900HT sequence detection system (Applied Biosystems). The relative expression levels of β-catenin mRNA were evaluated using the 2^−ΔΔCt^ method and normalized to glyceraldehyde 3-phosphate dehydrogenase (GAPDH). Primer sequences were as follows:

GAPDH.

Forward, 5’-GGACCTGACCTGCCGTCTAG-3’.

Reverse, 5’-GTAGCCCAGGATGCCCTTGA-3′.

β-catenin.

Forward, 5’-AAAGCGGCTGTTAGTCACTGG-3’.

Reverse, 5’-CGAGTCATTGCATACTGTCCAT-3′.

### Statistical analysis

All experimental results were repeated at least three times and are shown as mean ± standard deviation (s.d.). The association between CD36 expression level and clinico-pathological characteristics was statistically determined using the Pearson χ^2^ test. Differences between treated and control groups were analyzed using the Student’s *t*-test and one-way ANOVA. A two-tailed value of *P*<0.05 was considered statistically significant. All statistical analysis were performed with the Stata software 12.0 (Stata Corporation, College Station, TX, USA).

## Results

### CD36 is highly expressed in human GC tissues and associated with GC TNM staging and poor prognosis

**Fig. 1:**
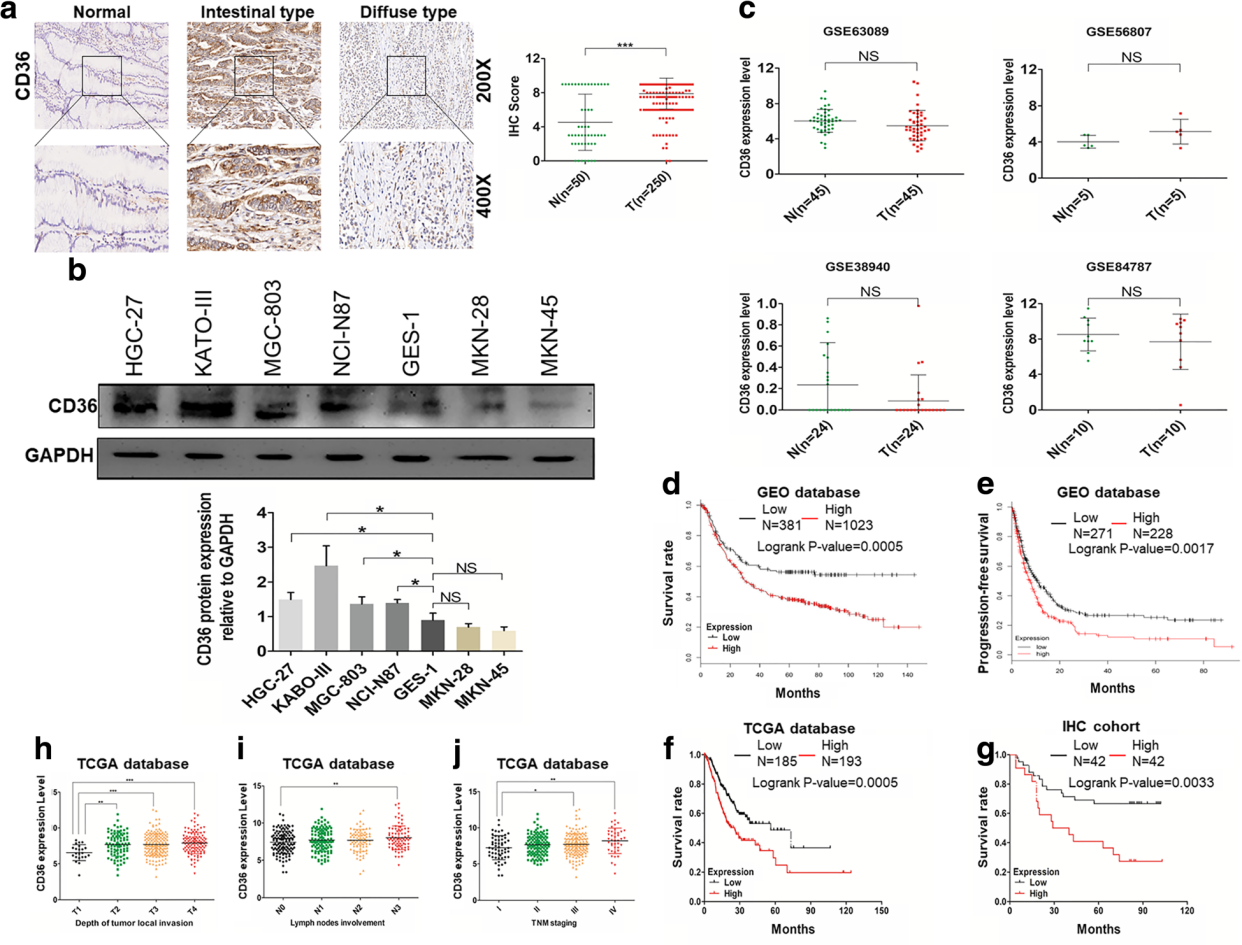
Expression of CD36 in GC tissues, normal tissues and cell lines and its correlation with the clinico-pathology of GC. **a** IHC staining of CD36 in GC tissues and adjacent non-tumor tissues. Histogram shows the IHC scores of CD36 expression in GC tissues and normal tissues. **b** The expression level of CD36 in GC cell lines and immortalized gastric epithelial cell line (GES-1) by western blot and densitometric measurements of CD36 protein levels. **c** CD36 mRNA levels in GC tissues and paired normal tissues from GEO database (T = GC tissues, *N* = paired normal tissues). **d**, **f** and **g** Cumulative survival curves of GC patients from GEO database, TCGA database and our own clinical data. **e** PPS analysis of GC patients from GEO database. **h**, **i** and **j** Correlation between CD36 expression in tumor tissues and clinico-pathological parameters based on TCGA database. *P<0.05, **P<0.01, ***P<0.001, ‘NS’ means not significant

To understand the role of CD36 in GC metastasis, we measured CD36 expression in GC tissues by IHC, and analyzed the relationship between the level of CD36 in GC tissues and patients’ clinico-pathological characteristics (validated by the TCGA GC cohort). Positive immunostaining of CD36 was mainly seen in the cytomembrane (Fig. 1a) and was significantly higher in GC tissues than in normal tissues. CD36 mRNA levels were similar in GC tissues and their paired normal gastric tissues using GEO datasets (Fig. 1c). The expression level of CD36 in tumor tissues was positively associated with depth of local tumor invasion (*P* = 0.043) and higher TNM stage (*P* = 0.0019), but not with other clinico-pathological parameters including age, tumor differentiation degree and tumor size (Table 1). We further validated these results using the TCGA database which showed that the expression level of CD36 in tumor tissues was also significantly associated with depth of local tumor invasion and higher TNM stage of GC, and also with lymph node involvement (Fig. 1h, i and j). In addition, Kaplan-Meier survival analysis showed that patients with high CD36 expression level in tumor tissues had poorer prognosis than those with lower CD36 expression using our own cohort (*P* = 0.0033) (Fig. 1g). This result was further validated by GEO datasets using Kaplan-Meier Plotter online (*P* = 0.0005) (Fig. 1d) and TCGA cohort (*P* = 0.0005) (Fig. 1f). We further investigated the correlation between the post-progression survival (PPS) of GC patients and CD36 expression in GC tissues. PPS is a more meaningful way to evaluate the GC metastatic potential and we found that CD36 expression was negatively correlated with GC patients’ PPS (*P* = 0.0017, Fig. 1e). CD36 protein levels in most GC cell lines (HGC27, KATO-III, MGC803, NCI-N87) were also significantly higher than in the immortalized gastric epithelial cell line (GES-1) (Fig. 1b). These results suggested that CD36 might play a key role in GC progression.

**Table 1:** Relationship between CD36 expression and clinico-pathological variables in 250 GC tissues

Variables	Number of 1cases	CD36 immunostaining		*P* value
		Negative (*N* = 108)	Positive (*N* = 142)	
Age(years)				
≥ 60	111	51	60	0.1625
< 60	139	57	82	
Gender				
Male	182	73	109	0.1162
Female	68	35	33	
Tumor size(cm)				
≥ 5	129	56	73	1
< 5	121	52	69	
Differentiation				
Poorly, undifferentiated	115	49	66	0.8985
Well, Moderately	135	59	76	
Local invasion				
T1 + T2	84	44	40	0.043
T3 + T4	166	64	102	
Lymph node involvement				
NO	65	28	37	1
YES	185	80	105	
TNM stage				
I,II	47	30	17	0.0019
III,IV	203	78	125	

### Palmitate acid promotes migration and invasion of GC cells

**Fig. 2:**
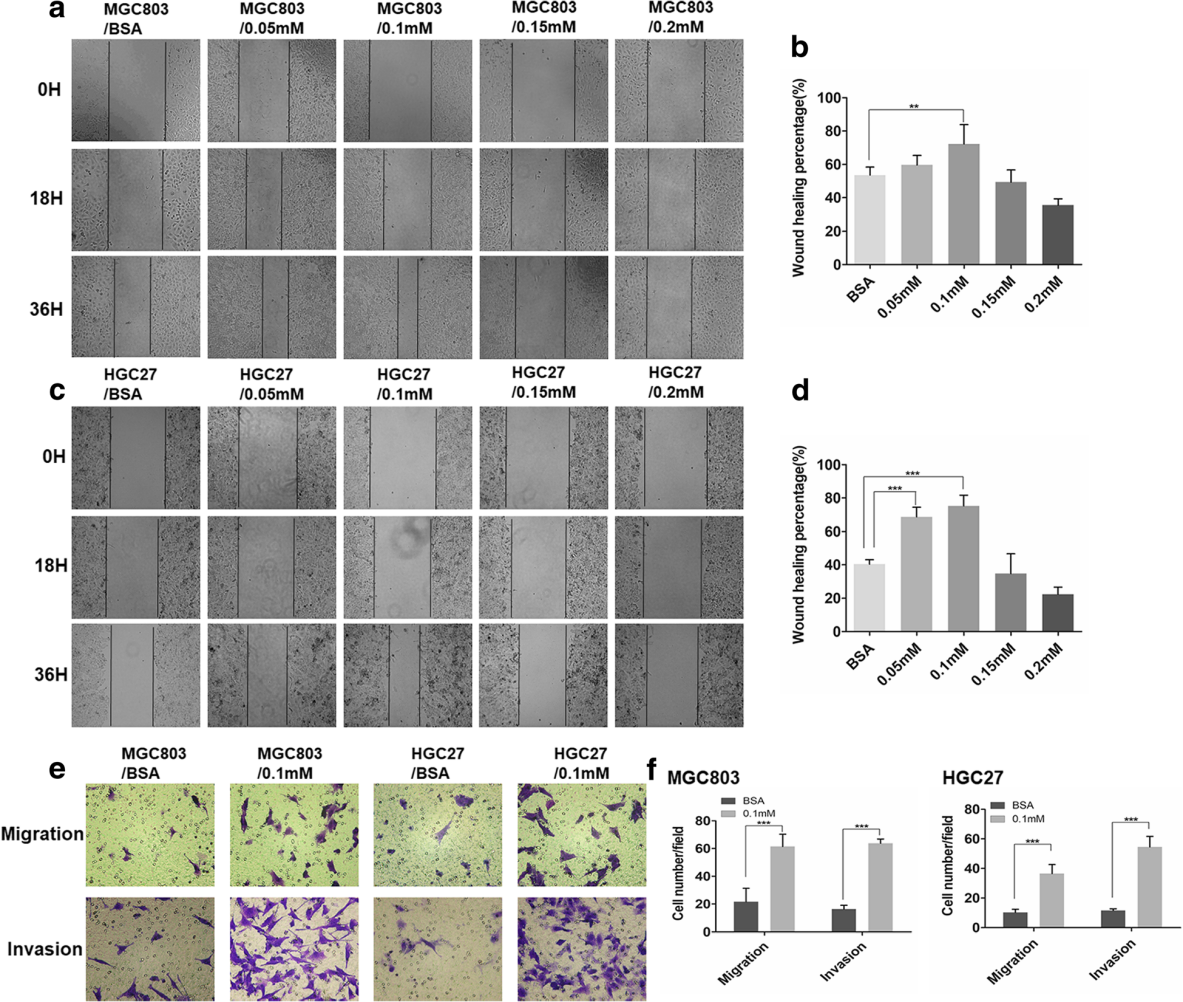
PA enhances the migratory and invasive abilities of GC cells. **a** and **c** Effect of different concentrations of PA on GC cell wound-healing (%) (mag. × 40). **b** and **d** Histograms of wound-healing (%) (mag. × 40) measured at 0, 18, and 36 h. **e** Effect of PA on GC cell migration and invasion (mag. × 200). **f** Histograms of the number of migrated and invaded cells (mag. × 200). Five random fields were selected for statistical analysis. Data are shown as mean ± SD of three independent experiments. *P<0.05, **P<0.01, ***P<0.001

We chose the saturated fatty acids, SA and PA, the monounsaturated fatty acid, OA, and the polyunsaturated fatty acid, DHA, and investigated their effects on the metastatic properties of GC cells. Firstly, to evaluate the influence of PA and SA on GC cells, we performed wound-healing assays, migration and invasion assays. PA promoted GC cell migration and wound-healing at an optimal concentration of 0.1 mM (Fig. 2a-d) while SA exhibited tumor-suppressing effects (Additional file 2: Figure S1a and b), indicating PA might participate in GC metastasis toward omentum. Further migration and invasion assays confirmed this result (Fig. 2e and f). The biological effects of the monounsaturated fatty acid, OA, and the polyunsaturated fatty acid, DHA, on GC metastasis had been previously demonstrated [7, 8]; and when we repeated the migration and invasion assays using the recommended concentrations of OA and DHA, we found that 100 μM OA significantly promoted metastasis of GC cell lines while 20 μM DHA inhibited metastasis of GC cell lines (Additional file 2: Figure S1c and d). Our observation that the saturated fatty acid, PA, could promote migration and invasion of GC cell lines had not been seen before, and therefore, PA was chosen for further analysis.

### CD36-mediated, PA-induced promotion of GC cell migration and invasion

**Fig. 3:**
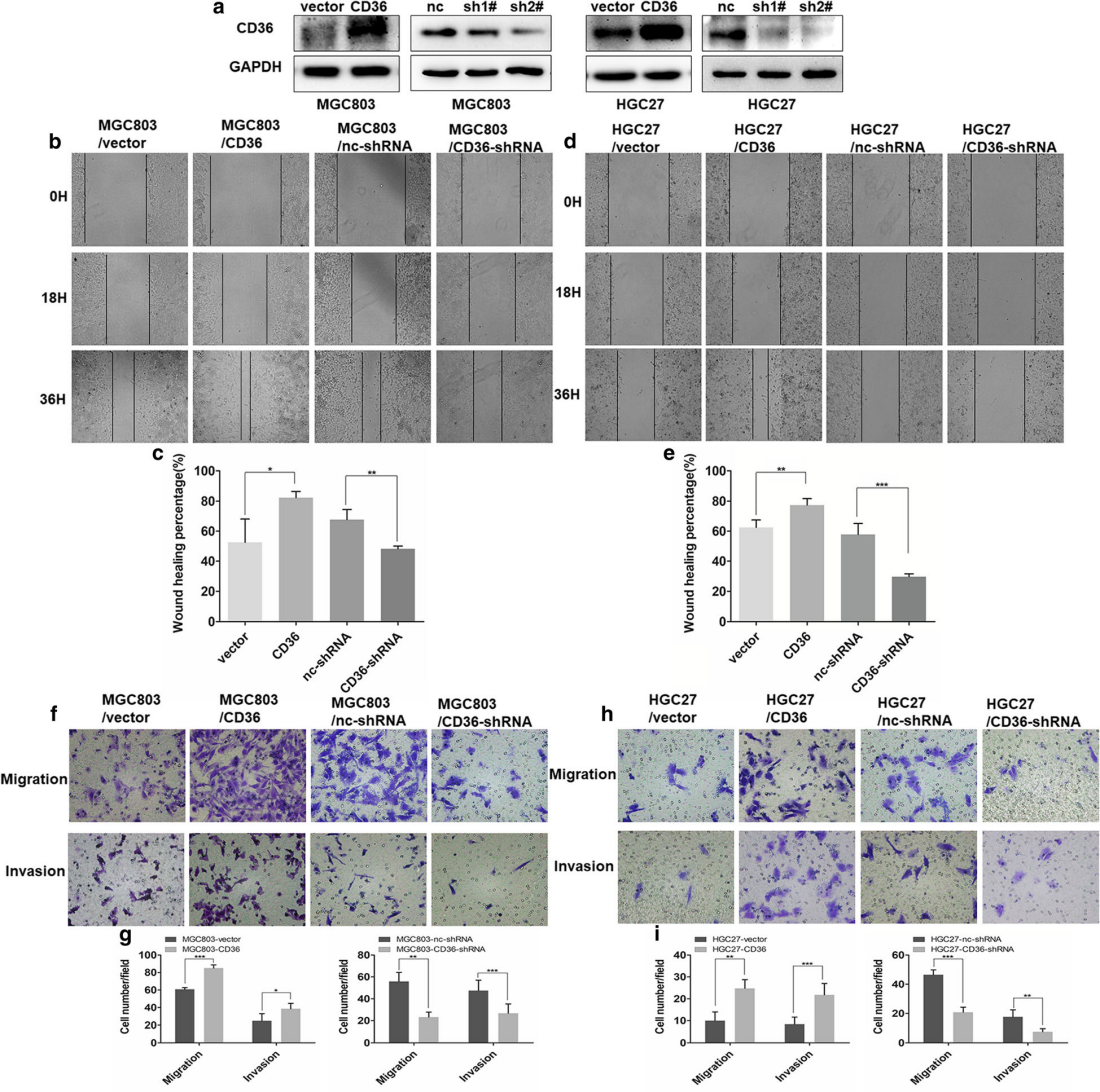
CD36 mediates the migration and invasion of GC cells induced by PA. **a** Western blot analysis of CD36 expression in MGC803 and HGC27 cells transfected with CD36-plasmid, vector-plasmid control, nc-shRNA plasmid, CD36-shRNA#1 plasmid or CD36-shRNA#2 plasmid. **b**, **d**, **f** and **h** Effect of CD36 knockdown or overexpression on wound-healing of GC cells (mag. × 40), migration and invasion (mag. × 200). **c**, **e**, **g** and **i** Histograms of wound-healing (%) (mag. × 40) and the number of migrated and invaded cells (mag. × 200). Five random fields were selected for statistical analysis. Data are shown as mean ± SD of three independent experiments. *P<0.05, **P<0.01, ***P<0.001

Because CD36 is at the top of the signaling cascade that removes lipids from the extra-cellular environment, we chose to determine whether the tumor-promoting effects of PA were mediated by CD36 by assaying for wound-healing, cell migration and invasiveness. Cells were stably transfected with plasmids overexpressing CD36 or silencing CD36 gene expression: MGC803/vector, MGC803/CD36, HGC27/vector, HGC27/CD36, MGC803/nc-shRNA, MGC803/CD36-shRNA, HGC27/nc-shRNA and HGC27/CD36-shRNA. CD36 expression and knock-down were verified by western blot (Fig. 3a). When incubated with 0.1 mM PA, the wound healed faster in MGC803/CD36 and HGC27/CD36 cells than in controls, MGC803/vector and HGC27/vector cells. Wound-healing was also more rapid in MGC803/nc-shRNA and HGC27/nc-shRNA cells than in the CD36 knock-down cell lines, MGC803/CD36-shRNA and HGC27/CD36-shRNA, treated with 0.1 mM PA (Fig. 3b-e). After treatment with 0.1 mM PA, CD36 knock-down MGC803 and HGC27 cells showed significantly reduced migration and invasion, while overexpression of CD36 markedly increased their migratory and invasive abilities (Fig. 3f-i). To further confirm the biological effects of CD36 in GC, we transfected the GC cell line, MKN28 (comparatively lower expression level of CD36 among GC cell lines) with the CD36 expression plasmid and its control, verified by western blot (Additional file 3: Figure S2a). As with the previous cell lines, treatment of MKN28 overexpressing CD36 with 0.1 mM PA, significantly increased its migratory and invasive properties, which was not found in BSA-treated control cells (Additional file 3: Figure S2b). Therefore, the evidence is consistent with CD36 playing a key role in PA-induced promotion of GC cell migration and invasion.

### CD36 promotes GC cell migration and invasion via uptake of exogenous palmitate acid

**Fig. 4:**
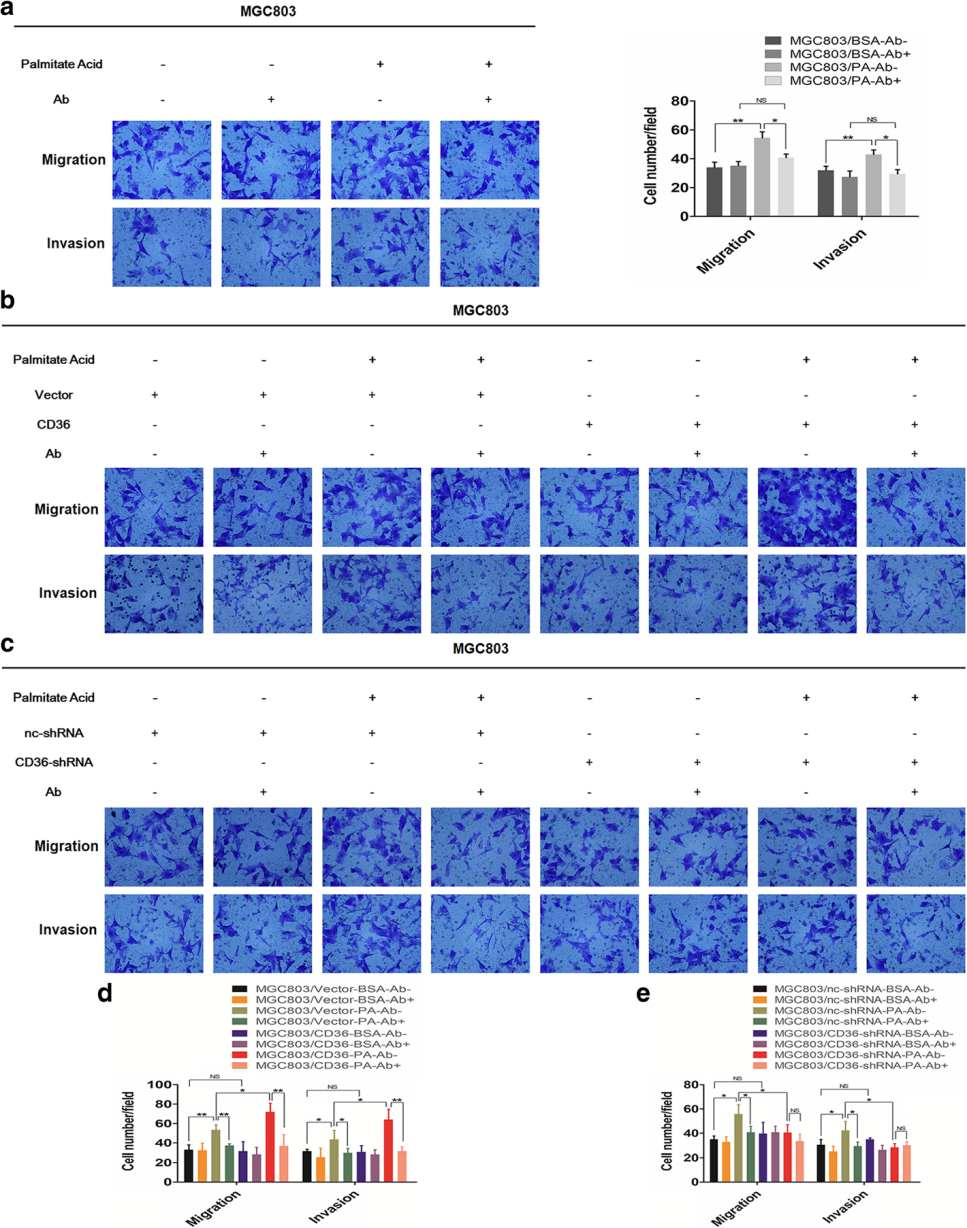
CD36 promotes GC metastasis via uptake of PA. **a** Specific block of cellular FA uptake with anti-CD36 antibody inhibits PA-mediated migration and invasion of MGC803. Histogram shows the number of migrated and invaded cells (mag. × 200). Five random fields were selected for statistical analysis. **b** and **c** Block of cellular FA uptake by anti-CD36 inhibited migration and invasion of PA-treated GC cells (MGC803/Vector, MGC803/CD36, MGC803/nc-shRNA, MGC803/CD36-shRNA). **d** and **e** Histograms of the number of migrated and invaded cells (mag. × 200). Five random fields were selected for statistical analysis. Data are shown as mean ± SD, *P<0.05, **P<0.01, ***P<0.001, ‘NS’ means not significant

To ascertain whether CD36 promoted GC metastasis mainly through mediating the uptake of exogenous PA, we prevented FA uptake by blocking the CD36 receptor with an anti-CD36 neutralizing antibody (JC63.1), with the IgA isotype as the control [24]. Blocking with anti-CD36 significantly inhibited the metastasis-promoting effect of PA (Fig. 4a, Additional file 4: Figure S3a). We also found no significant difference between MGC803/Vector and MGC803/CD36, MGC803/nc-shRNA and MGC803/CD36-shRNA, HGC27/vector and HGC27/CD36, or HGC27/nc-shRNA and HGC27/CD36-shRNA in the absence of PA treatment (Fig. 4b-e, Additional file 4: Figure S3b-e), which means that the CD36-mediated effects are dependent on PA. Anti-CD36 decreased the tumor-promoting effects of PA in GC cells with basal level or overexpression of CD36 but not with silenced CD36 (Fig. 4b-e, Additional file 4: Figure S3b-e), further supporting the hypothesis that CD36 contributed to the metastasis of GC by taking up exogenous PA.

### Palmitate acid induces GC migration and invasion through CD36-dependent activation of the AKT pathway

**Fig. 5:**
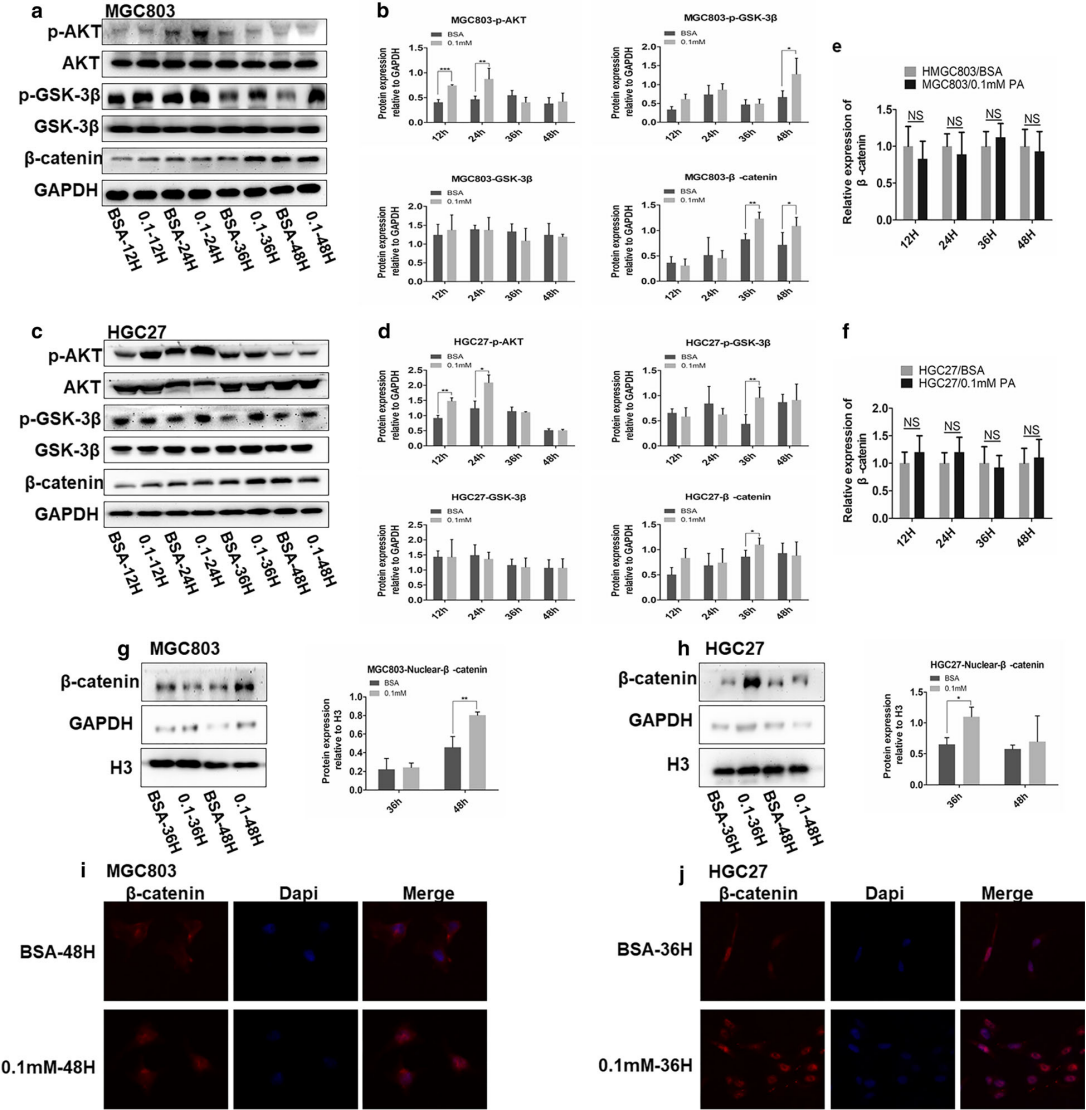
PA promotes GC cell migration and invasion via the AKT/GSK-3β/ β-catenin signaling pathway. **a** and **c** Effect of PA on protein levels of p-AKT, p-GSK-3β, AKT, GSK-3β and global β-catenin at different time points. **b** and **d** Densitometry showing effect of PA on protein levels of p-AKT, p-GSK-3β, AKT, GSK-3β and global β-catenin. **e** and **f** Effect of PA on cellular mRNA level of β-catenin at different time points. **g** and **h** Effects of PA on nuclear-transport of β-catenin at selected time points. Densitometry showing effect of PA on nuclear protein level of β-catenin. **i** and **j** IF showing effect of PA on cellular location of β-catenin in GC cells at selected time points. Data are shown as mean ± SD of three independent experiments. *P<0.05, **P<0.01, ***P<0.001, ‘NS’ means not significant

To explore the mechanism of PA-mediated metastasis of GC, we surveyed several proteins known to be associated with tumor metastasis, and found that nuclear accumulation of β-catenin might be involved in PA-induced tumor metastasis. β-catenin over-expression is associated with many types of cancer and has been reported to play an important role in cancer metastasis [25, 26]. The degradation of β-catenin is mediated by the phosphokinase activity of GSK-3β [27–29], which is inhibited through phosphorylation by activated phospho-AKT, another classic oncogenic kinase playing a remarkable role in tumor progression [30–33]. Hence, we measured the protein level of p-AKT, total AKT, p-GSK-3β, total GSK-3β and β-catenin by western immunoblot analysis. We determined that 0.1 mM PA increased the level of phospho-AKT and β-catenin as well as phospho-GSK-3β while the levels of total AKT and GSK-3β protein remained unchanged (Fig. 5a-d). IF assay and nuclear-plasma separation assay proved that PA significantly increased the nuclear localization of β-catenin, which acted as a transcription factor functioning within nucleus (Fig. 5g-j). We also performed qRT-PCR analysis to quantitate the mRNA level of β-catenin in MGC803 and HGC27 cells treated with either 0.1 mM PA or BSA at different times and found that PA did not alter the transcriptional level of β-catenin compared to its controls in GC cell lines (Fig. 5e and f), which further proved that PA promoted nuclear transport and accumulation of β-catenin by inhibiting its degradation by GSK-3β.

**Fig. 6:**
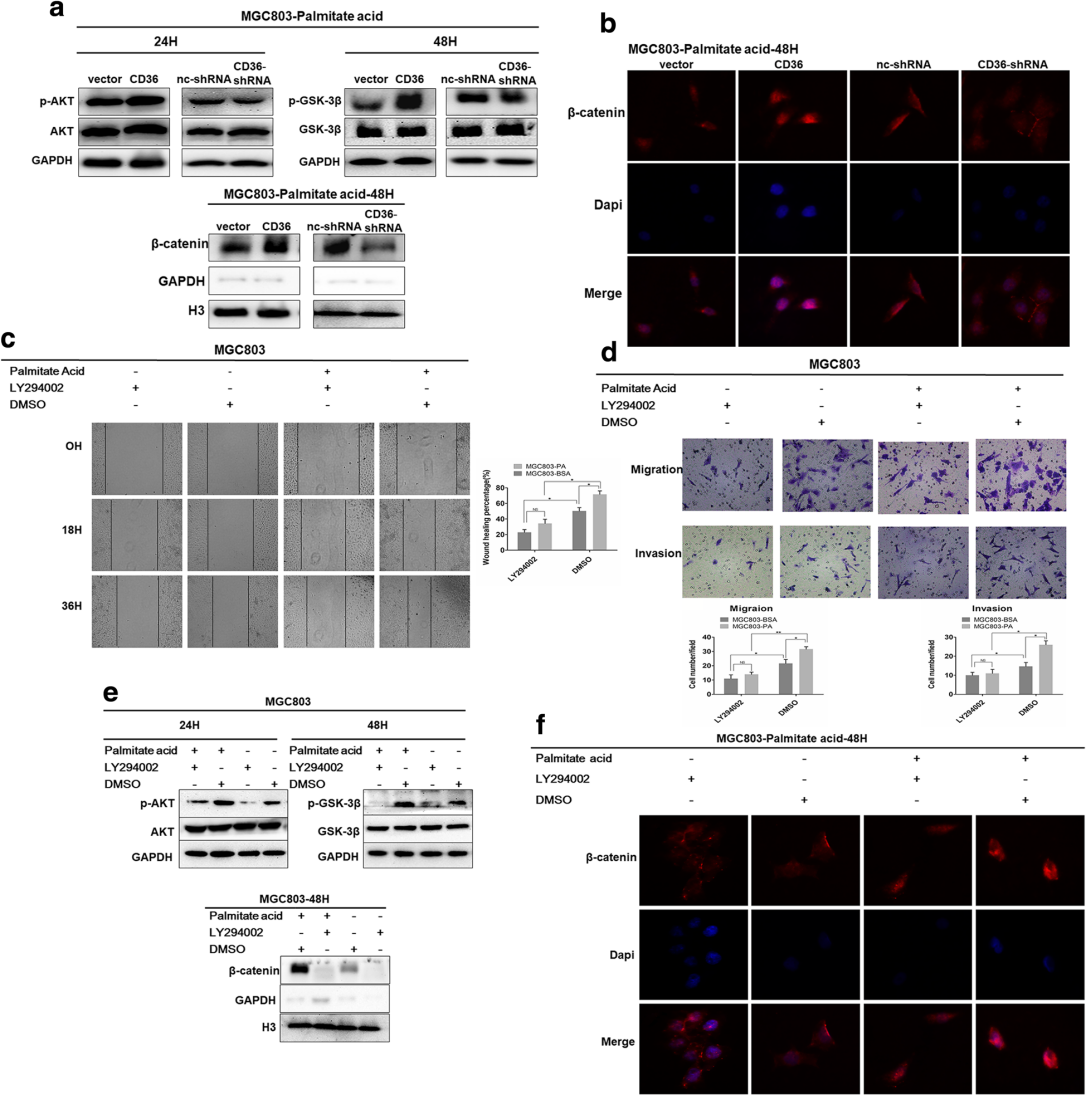
CD36 mediates PA-induced activation of AKT/GSK-3β/β-catenin signaling. **a** Effects of knockdown and overexpression of CD36 on p-AKT, AKT, p-GSK-3β, GSK-3β and nuclear β-catenin. **b** IF showing effects of knockdown and overexpression of CD36 on cellular location of β-catenin in PA-treated GC cells. **c** and **d** PI3K inhibitor, LY294002 inhibited migration and invasion of MGC803. Histograms show wound-healing (%) (mag. × 40) and number of migrated and invaded cells (mag. × 200). Five random fields were selected for statistical analysis. **e** Expression levels of p-AKT, AKT, p-GSK-3β, GSK-3β, nuclear β-catenin in PA-treated MGC803 in the presence of LY294002, compared to controls. **f** IF showing effects of LY294002 on cellular location of β-catenin in PA-treated GC cells, compared to controls. Data are shown as mean ± SD of three independent experiments. *P<0.05, **P<0.01, ***P<0.001, ‘NS’ means not significant

In order to verify the role of CD36 in AKT activation and the nuclear localization of β-catenin caused by PA, we performed western blot analyses that demonstrated that CD36 knockdown significantly reduced the phosphorylation level of both AKT and GSK-3β as well as the nuclear localization of β-catenin, while overexpression of CD36 had the opposite effect compared with the control groups when treated with 0.1 mM PA (Fig. 6a, b, Additional file 5: Figure S4a and b).

To further investigate whether the tumor-promoting effects induced by PA depended on activation of the AKT pathway, we used the PI3K-specific inhibitor, LY294002 (10 μM) and then tested wound-healing, migration and invasion. We found that PA significantly promoted migration and invasion of GC cells when treated with vehicle (DMSO), but not when GC cells were treated with 10 μM LY294002 (Fig. 6c and d, Additional file 5: Figure S4c and d). PA also significantly elevated the protein levels of p-AKT, p-GSK-3β and the nuclear localization of β-catenin in cells treated with DMSO, while these effects were reversed by 10 μM LY294002 (Fig. 6e and f; Additional file 5: Figure S4e and f). These results indicated that the PA-induced tumor-promoting effects depended on activation of the AKT pathway.

### Fatty acids acting through CD36 facilitated growth of peritoneally implanted GC cells in vivo

**Fig. 7:**
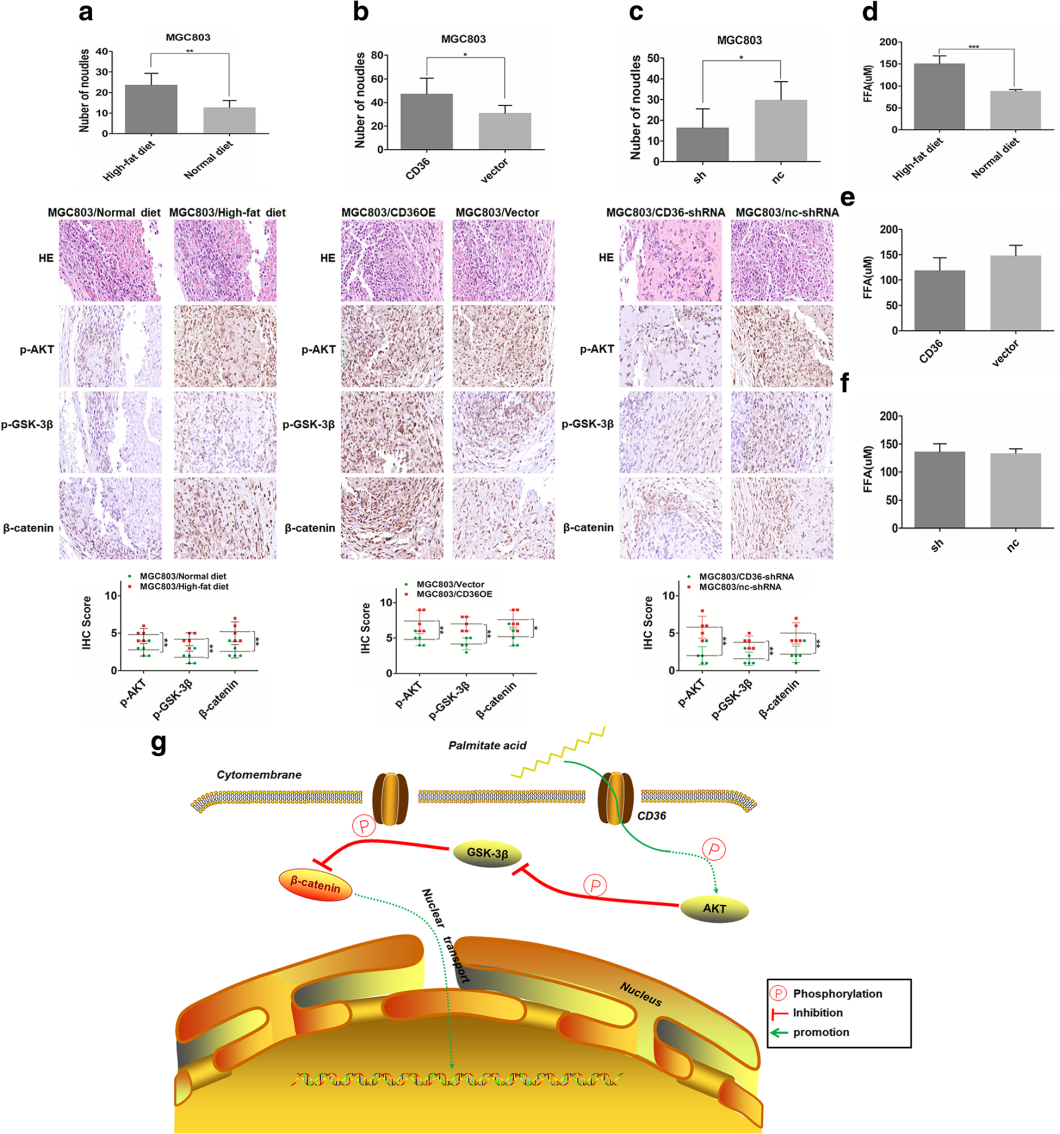
CD36 mediates PA-induced peritoneal dissemination in nude mice. **a**, **b** and **c** (*upper*) histograms of peritoneal tumor nodules four weeks after implantation of tumor cells (MGC803, MGC803/vector, MGC803/CD36, MGC803/nc-shRNA, MGC803/CD36-shRNA) into the peritoneum of nude mice. (*middle*) Histological appearance of disseminated tumors in the abdominal cavities of each group by H&E staining and IHC of p-AKT, p-GSK-3β, and β-catenin. (mag. × 400). (*lower*) Histograms show the IHC scores of p-AKT, p-GSK-3β and β-catenin in tumor tissues of each group. **d**, **e** and **f** Concentration of free long-chain FAs in the serum of mice. **g** CD36 mediates cellular uptake of PA which activates AKT through phosphorylation and then inactivates GSK-3β, which inhibits degradation of β-catenin and promotes its nuclear-transportation. Data are shown as mean ± SD. *P<0.05, **P<0.01, ***P<0.001

In order to evaluate whether FAs could promote metastasis of a gastric tumor and whether this process was mediated by CD36 in vivo, a peritoneal dissemination assay was performed in nude mice. As shown in Fig. 7a, there were significantly fewer visible peritoneal nodules in the MGC803/Normal diet group compared with MGC803/high-fat diet group. We measured the concentration of long-chain FAs in the serum of the mice and found that they were higher in mice fed the high-fat diet relative to the control group (Fig. 7d), indicating that long-chain FAs were available to promote the growth of peritoneally implanted GC cells. Mice implanted with CD36-overexpressing cells, MGC803/CD36, and fed with the high fat diet, showed even greater numbers of peritoneal metastatic nodules compared with mice implanted with control cells, MGC803/vector (Fig. 7b), while the group implanted with MGC803/CD36-shRNA cells showed significantly fewer visible peritoneal nodules compared to its control group (Fig. 7c). The concentration of long-chain FAs in the serum of these four groups was similar (Fig. 7d-f). A histological analysis of the xenografts confirmed this result. We performed IHC assays to measure the levels of specific protein in tumor nodules of each group. The nodules of the high-fat diet group consistently exhibited higher protein levels of p-AKT and p-GSK-3β and β-catenin compared to the normal diet group (Fig. 7a). Up-regulation of CD36 amplified the FA-induced biological effects (Fig. 7b), while CD36 down-regulation inhibited the effects (Fig. 7c). These results indicated that long-chain FAs could promote the growth of peritoneally implanted GC cells in vivo through uptake by CD36.

## Discussion

FAs, including both saturated fatty acids and unsaturated fatty acids have recently been reported to play a vital role in the initiation and progression of multiple types of cancer both in vitro and in vivo [6, 23, 34, 35]. Considering the fact that GC has a clear predilection for metastasis toward omentum which is primarily composed of adipose tissue, we hypothesized that FAs may play an indispensable role in the peritoneal metastasis of GC. FAs include saturated fatty acids, monounsaturated fatty acids and polyunsaturated fatty acids. Given that the effects of OA (monounsaturated FA) and DHA (polyunsaturated FA) on GC cells in our study were similar to published results [7, 8], we then chose to investigate the biological effects of saturated FAs on GC. Based on comparative studies, it is known that the saturated FA, stearic acid (SA) exhibits tumor-suppressing effects while palmitate acid (PA) has tumor-promoting effects. PA is one of the major FAs in human blood, therefore, PA was utilized in our research to investigate the biological function of saturated FAs in the metastasis of GC. PA is a type of C16 saturated FA which has been reported to facilitate the metastasis of oral carcinomas [23], but the connection between PA and GC has not been researched. Here we demonstrated that PA promoted both migration and invasion of GC through activation of AKT. Previous research had shown that the accumulation of free FAs (FFAs), especially the saturated FFA, palmitate acid, led to mitochondrial dysfunction and generation of reactive oxidative species (ROS) that contributed to loss of cellular homeostasis and cell death, a phenomenon termed lipotoxicity [36]. In our study we also found that high concentrations of PA inhibited GC migratory and invasive abilities possibly due to lipotoxicity, so in our experiments we chose the nontoxic working concentration of 0.1 mM. We found that PA activated AKT through phosphorylation, which then resulted in the inactivation of GSK-3β via phosphorylation [30–32, 37]. Previous studies proved that activation of AKT led to phosphorylation and inactivation of GSK-3β which inhibited degradation of β-catenin [25–27, 29]. We determined that PA indeed promoted the nuclear localization of β-catenin while exhibiting no influence on its mRNA level. Additionally, the PI3K inhibitor, LY294002, significantly inhibited the phosphorylation of AKT and GSK-3β which consequently decreased the nuclear localization of β-catenin and therefore suppressed the tumor-promoting effects of PA. These results emphasize the paramount role of AKT/GSK-3β/β-catenin signaling in PA-induced metastasis of GC. However, PA has multiple biological functions and could positively or negatively control other signaling pathways. The overall influence of PA on the AKT/GSK3-β/β-catenin signaling pathway is not straightforward and may vary with different cell types; thus, the exact mechanism of PA action remains to be clarified.

CD36, a cell surface receptor, mediates the uptake of exogenous FAs including PA [9–12], and has been demonstrated to contribute to the initiation and progression of multiple types of cancers [13, 23]. We found that CD36 protein levels were significantly higher in GC tissues than in normal tissues. However, CD36 mRNA levels were not significantly different between paired GC tissues and normal tissues based on four microassays from the GEO database. This result may be due to posttranscriptional mediation by non-coding RNA or variation in tumor density. We then demonstrated that the expression level of CD36 in GC tissues closely correlated with TNM stage and with patients’ prognosis using TCGA database, GEO database and our own clinical data. We performed PPS analysis of CD36 using the GEO database because PPS analysis is a more meaningful way to evaluate GC metastatic ability. Through mediating the uptake of exogenous FAs, CD36 acts as a crucial component in the regulation of PA-induced metastasis of GC. Our results demonstrate that CD36 may play an overwhelming role in the biological processes induced by PA, which is supported by the evidence that upregulation of CD36 promoted migration and invasion of GC while knockdown of CD36 exhibited the opposite effect via AKT/GSK-3β/β-catenin signaling after treatment with PA. To test whether CD36 promoted GC metastasis mainly through mediating the uptake of exogenous PA, we performed blocking assays and found that CD36 indeed contributed to the migration and invasion of GC via mediating the uptake of exogenous PA.

To determine whether PA and CD36 participated in the metastasis of GC in vivo, peritoneal dissemination assay is performed as peritoneum is the most common metastatic site [19] and the most common site of recurrence which markedly influences GC patients’ survival [38]. In addition, peritoneum is mainly composed of adipose tissue, thus, peritoneal tumor dissemination is more applicable as we investigate the biological effects of fatty acids on GC metastasis. We fed one group of nude mice with a high-fat diet one week before inoculating them with GC tumor cells and another group with normal diet as control. The high-fat diet promoted the growth of peritoneally implanted GC cells via CD36 uptake of FAs. Upregulation of CD36 amplified the in vivo biological effects of FAs while knockdown of CD36 decreased them. Histological analysis confirmed this result. IHC measurement of protein levels of p-AKT, p-GSK-3β, and β-catenin showed that the high-fat diet did indeed elevate cellular protein levels of p-AKT, p-GSK-3β and the nuclear protein levels of β-catenin in GC via CD36.

## Conclusion

Our results are consistent with the model depicted in Fig. 7g. PA entering into GC cells via its receptor CD36, activates AKT through phosphorylation, which then inactivates GSK-3β via phosphorylation. Inactivation of GSK-3β decreases the degradation of β-catenin, which allows increased transport of β-catenin into the nucleus. Thus, through its role in mediating the uptake of exogenous FAs, CD36, as the surface receptor of FAs, mediates the tumor-promoting effect of PA both in vitro and in vivo. Our findings have further substantiated the significance of FAs in the metastasis of GC. We clearly established CD36 as an indispensable component of the PA-induced growth of peritoneally implanted GC. Therefore, drugs targeting CD36 might constitute a promising new treatment for peritoneal metastasis of GC.

## Additional files

Additional file 1: **Table S1.** List of antibodies involved. (DOCX 16 kb)

Additional file 2: **Figure S1.** Effect of SA, OA and DHA on metastasis of GC cells. (a) and (b) Effect of different concentrations of SA on GC cell wound-healing (%) (mag. × 40). Histograms show wound-healing (%) (mag. × 40) at 0, 18, and 36 h. (c) and (d) Effect of OA (100uM) and DHA (20 uM) on GC cell migration and invasion (mag. × 200). Histograms show number of migrated and invaded cells (mag. × 200). Five random fields were selected for statistical analysis. Data are shown as mean ± SD of three independent experiments. *P<0.05, **P<0.01, ***P<0.001. (TIF 7552 kb)

Additional file 3: **Figure S2.** Up-regulation of cellular CD36 expression significantly promoted migration and invasion of GC cell line MKN28. (a) CD36 expression in MKN28 cells transfected with plasmid over-expressing CD36 or vector-plasmid control. (b) Effect of CD36 overexpression on GC cell migration and invasion (mag. × 200). Histogram shows the number of migrated and invaded cells (mag. × 200). Five random fields were selected for statistical analysis. Data are shown as mean ± SD of three independent experiments. *P<0.05, **P<0.01, ***P<0.001, ‘NS’ means not significant. (TIF 3174 kb)

Additional file 4: **Figure S3.** CD36 promotes GC metastasis by cellular uptake of PA. (a) Blocking FA uptake with anti-CD36 antibody inhibits migration and invasion of PA-treated HGC27 cells compared to controls. Histogram shows number of migrated and invaded cells (mag. × 200). Five random fields were selected for statistical analysis. (b) and (c) Blocking FA uptake with anti-CD36 inhibits migration and invasion of PA-treated GC cells (HGC27/Vector, HGC27/CD36, HGC27/nc-shRNA, HGC27/CD36-shRNA) compared to controls. (d) and (e) Histograms of the number of migrated and invaded cells (mag. × 200). Five random fields were selected for statistical analysis. Data are shown as mean ± SD *P<0.05, **P<0.01, ***P<0.001, ‘NS’ means not significant. (TIF 13467 kb)

Additional file 5: **Figure S4.** CD36 mediates PA-induced activation of AKT/GSK-3β/β-catenin signaling. (a) Effects of knockdown and overexpression of CD36 on p-AKT, AKT, p-GSK-3β, GSK-3β and nuclear β-catenin. (b) Effects of knockdown and overexpression of CD36 on cellular location of β-catenin by IF of PA-treated GC cells compared to controls. (c) and (d) PI3K inhibitor, LY294002, reduces migration and invasion of HGC27 cells relative to controls. Histograms show wound-healing percentage (%) (mag. × 40) and the number of migrated and invaded cells (mag. × 200). Five random fields were selected for statistical analysis. (e) Expression of p-AKT, AKT, p-GSK-3β, GSK-3β and nuclear β-catenin in PA-treated HGC27 cells incubated with LY294002 relative to controls. (f) Effect of LY294002 on cellular location of β-catenin in PA-treated GC cells by IF, relative to controls. Data are shown as mean ± SD of three independent experiments. *P<0.05, **P<0.01, ***P<0.001. ‘NS’ means not significant. (TIF 10496 kb)

## Abbreviations

BSA: FA-free Bovine Serum Albumin
GC: Gastric cancer
GEO: Gene Expression Omnibus
GES-1: Immortalized normal gastric epithelial cell line
IHC: Immunohistochemical
PA: Palmitate acid
TCGA: The Cancer Genome Atlas
