# Supplementary material for: CD36 mediates palmitate acid-induced metastasis of gastric cancer via AKT/GSK-3β/β-catenin pathway
Source: J Exp Clin Cancer Res. 2019 Feb 4;38:52. doi: 10.1186/s13046-019-1049-7 (PMC6360779; doi:10.1186/s13046-019-1049-7)
Supplement: Supplementary file 1 — Table S1. List of antibodies involved. (DOCX 16 kb) [file 13046_2019_1049_MOESM1_ESM.docx]

**Additional File 5: Table S1.** List of antibodies involved.

| **Antibody** | **Company** | **Western blot** | **Immunohisto**  **chemistry** | **Immunofluorescence** | **Blocking** |
| --- | --- | --- | --- | --- | --- |
| p-AKT | CST(4070), Boston, USA | 1:1000 | NA | NA | NA |
| AKT | CST(4691), Boston, USA | 1:1000 | NA | NA | NA |
| p-GSK-3β | CST(5558), Boston, USA | 1:1000 | NA | NA | NA |
| GSK-3β | CST(12456), Boston, USA | 1:1000 | NA | NA | NA |
| β-catenin | CST(8480), Boston, USA | 1:1000 | NA | 1:200 | NA |
| CD36 | Abcam(ab133625), Cambridge, UK | 1:1000 | 1:200 | NA | NA |
| H3 | CST(4499), Boston, USA | 1:2000 | NA | NA | NA |
| GAPDH | Proteintech(HRP-60004), Rosemont, USA | 1:5000 | NA | NA | NA |
| Secondary antibody | Invitrogen(A-21207), Eugene, USA | NA | NA | 1:200 | NA |
| Secondary antibody | Proteintech(SA00001-2), Rosemont, USA | 1:5000 | NA | NA | NA |
| Secondary antibody | Proteintech(SA00001-1), Rosemont, USA | 1:5000 | NA | NA | NA |
| Anti-CD36 (JC63.1) | Abcam(ab23680), Cambridge, UK | NA | NA | NA | 1:500 |
| IgA | Abcam(ab37322), Cambridge, UK | NA | NA | NA | 1:500 |
