# Supplementary figures and images for: CD36 mediates palmitate acid-induced metastasis of gastric cancer via AKT/GSK-3β/β-catenin pathway
Source: J Exp Clin Cancer Res. 2019 Feb 4;38:52. doi: 10.1186/s13046-019-1049-7 (PMC6360779; doi:10.1186/s13046-019-1049-7)

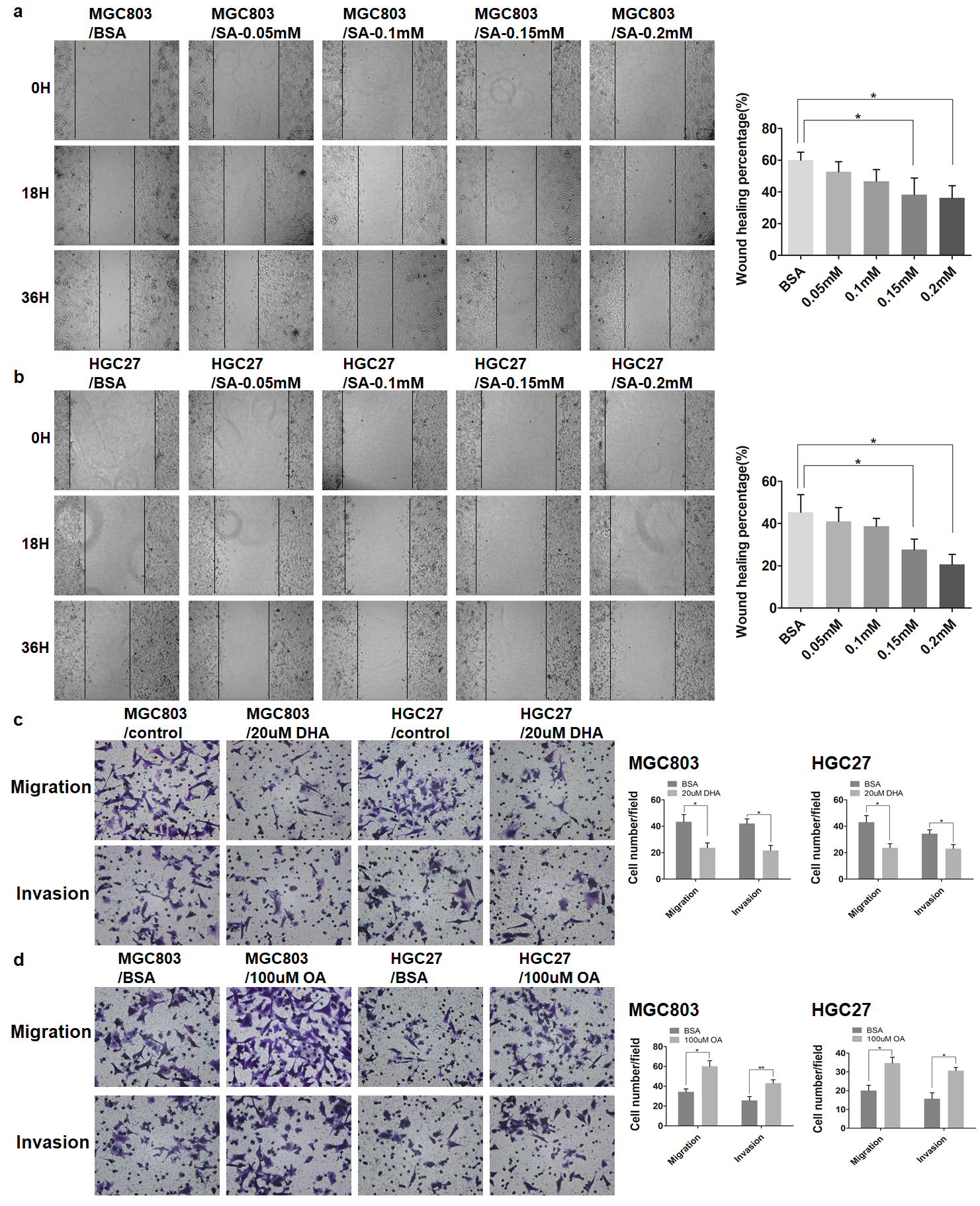

Supplement: Supplementary file 2 — Figure S1. Effect of SA, OA and DHA on metastasis of GC cells. (a) and (b) Effect of different concentrations of SA on GC cell wound-healing (%) (mag. × 40). Histograms show wound-healing (%) (mag. × 40) at 0, 18, and 36 h. (c) and (d) Effect of OA (100uM) and DHA (20 uM) on GC cell migration and invasion (mag. × 200). Histograms show number of migrated and invaded cells (mag. × 200). Five random fields were selected for statistical analysis. Data are shown as mean ± SD of three independent experiments. *P<0.05, **P<0.01, ***P<0.001. (TIF 7552 kb) [file 13046_2019_1049_MOESM2_ESM.tif]

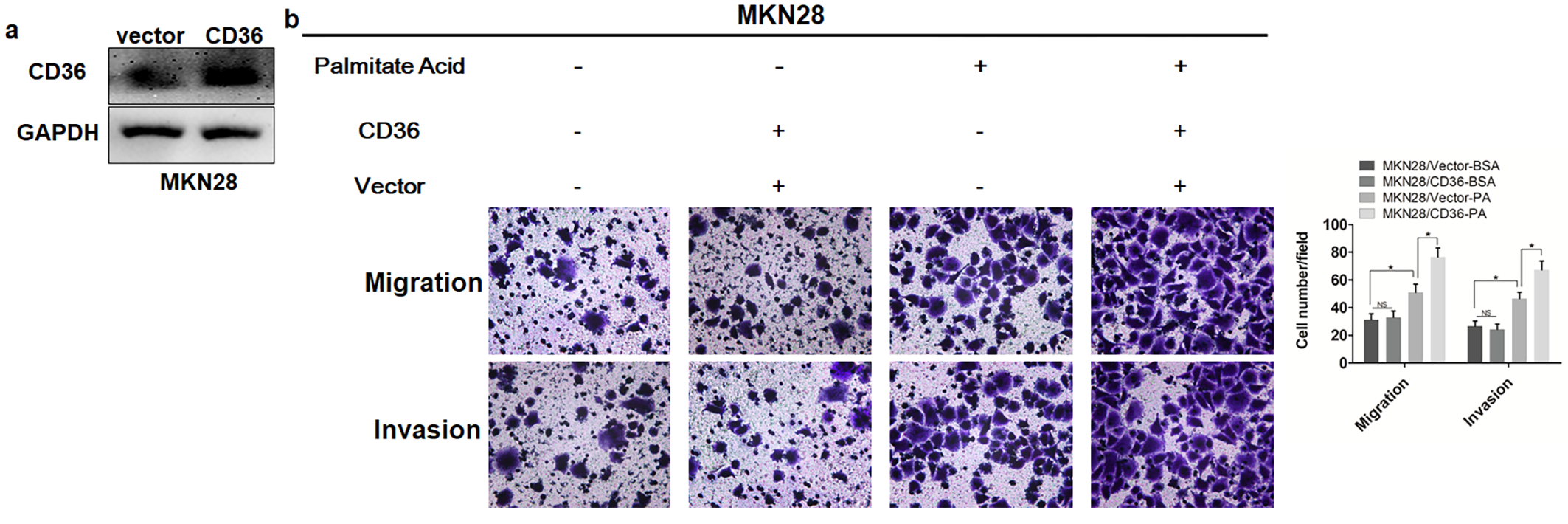

Supplement: Supplementary file 3 — Figure S2. Up-regulation of cellular CD36 expression significantly promoted migration and invasion of GC cell line MKN28. (a) CD36 expression in MKN28 cells transfected with plasmid over-expressing CD36 or vector-plasmid control. (b) Effect of CD36 overexpression on GC cell migration and invasion (mag. × 200). Histogram shows the number of migrated and invaded cells (mag. × 200). Five random fields were selected for statistical analysis. Data are shown as mean ± SD of three independent experiments. *P<0.05, **P<0.01, ***P<0.001, ‘NS’ means not significant. (TIF 3174 kb) [file 13046_2019_1049_MOESM3_ESM.tif]

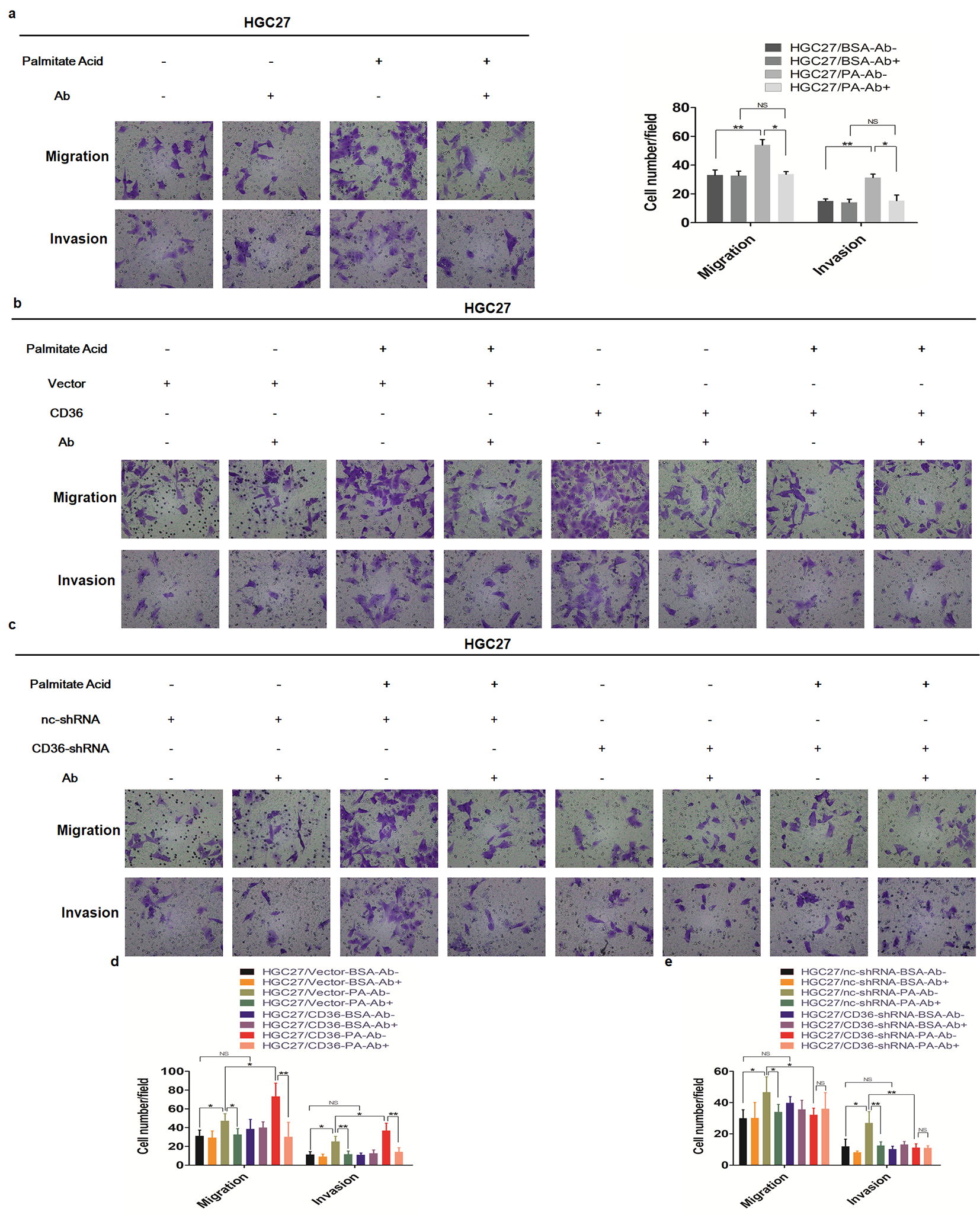

Supplement: Supplementary file 4 — Figure S3. CD36 promotes GC metastasis by cellular uptake of PA. (a) Blocking FA uptake with anti-CD36 antibody inhibits migration and invasion of PA-treated HGC27 cells compared to controls. Histogram shows number of migrated and invaded cells (mag. × 200). Five random fields were selected for statistical analysis. (b) and (c) Blocking FA uptake with anti-CD36 inhibits migration and invasion of PA-treated GC cells (HGC27/Vector, HGC27/CD36, HGC27/nc-shRNA, HGC27/CD36-shRNA) compared to controls. (d) and (e) Histograms of the number of migrated and invaded cells (mag. × 200). Five random fields were selected for statistical analysis. Data are shown as mean ± SD *P<0.05, **P<0.01, ***P<0.001, ‘NS’ means not significant. (TIF 13467 kb) [file 13046_2019_1049_MOESM4_ESM.tif]

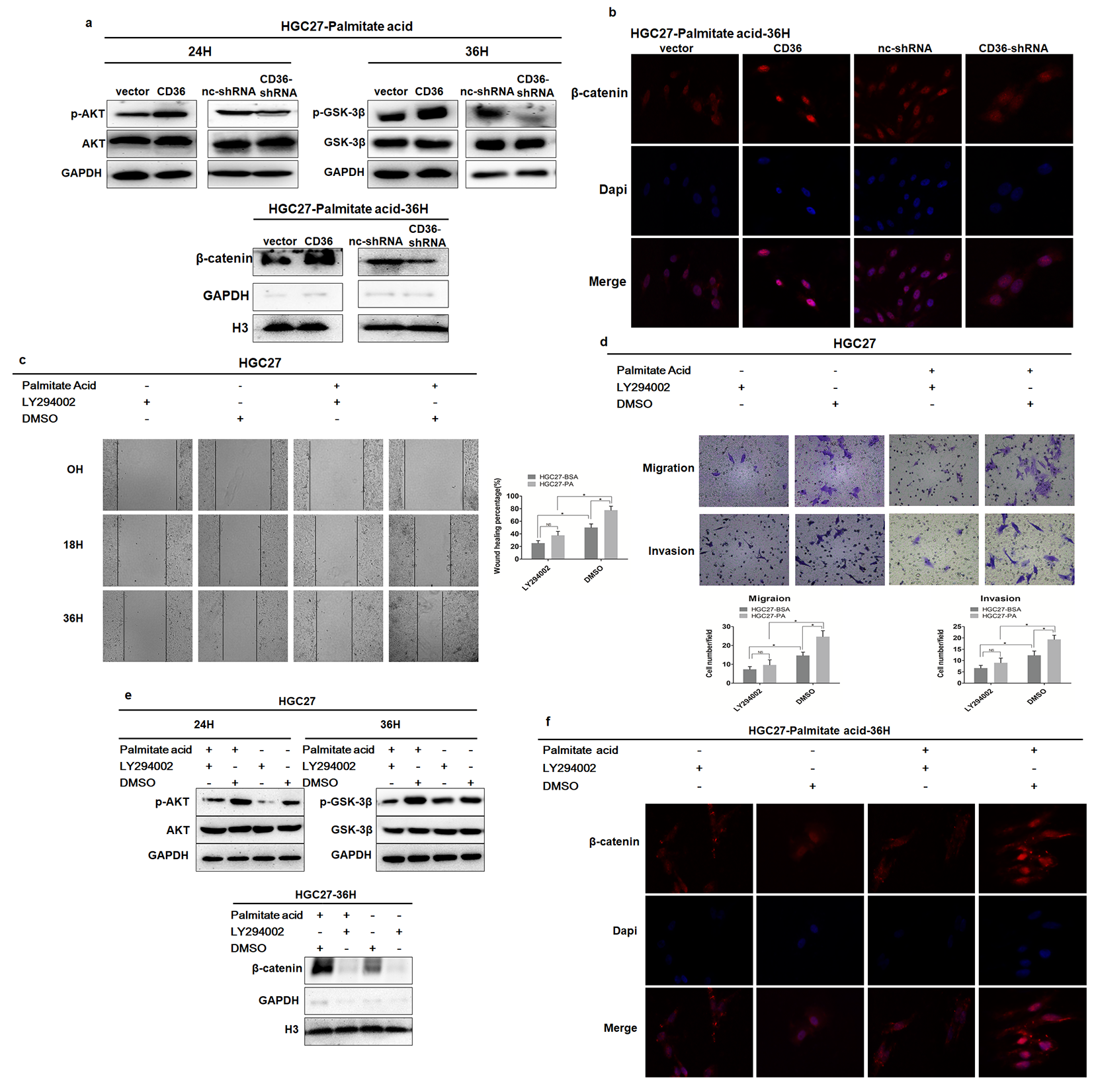

Supplement: Supplementary file 5 — Figure S4. CD36 mediates PA-induced activation of AKT/GSK-3β/β-catenin signaling. (a) Effects of knockdown and overexpression of CD36 on p-AKT, AKT, p-GSK-3β, GSK-3β and nuclear β-catenin. (b) Effects of knockdown and overexpression of CD36 on cellular location of β-catenin by IF of PA-treated GC cells compared to controls. (c) and (d) PI3K inhibitor, LY294002, reduces migration and invasion of HGC27 cells relative to controls. Histograms show wound-healing percentage (%) (mag. × 40) and the number of migrated and invaded cells (mag. × 200). Five random fields were selected for statistical analysis. (e) Expression of p-AKT, AKT, p-GSK-3β, GSK-3β and nuclear β-catenin in PA-treated HGC27 cells incubated with LY294002 relative to controls. (f) Effect of LY294002 on cellular location of β-catenin in PA-treated GC cells by IF, relative to controls. Data are shown as mean ± SD of three independent experiments. *P<0.05, **P<0.01, ***P<0.001. ‘NS’ means not significant. (TIF 10496 kb) [file 13046_2019_1049_MOESM5_ESM.tif]
